# Neonatal Immunity, Respiratory Virus Infections, and the Development of Asthma

**DOI:** 10.3389/fimmu.2018.01249

**Published:** 2018-06-04

**Authors:** Katherine H. Restori, Bharat T. Srinivasa, Brian J. Ward, Elizabeth D. Fixman

**Affiliations:** ^1^Research Institute of the McGill University Health Centre, Montréal, QC, Canada; ^2^Meakins-Christie Laboratories, Research Institute of the McGill University Health Centre, Montréal, QC, Canada

**Keywords:** asthma, neonatal immune system, respiratory infections, respiratory syncytial virus, influenza, rhinovirus

## Abstract

Infants are exposed to a wide range of potential pathogens in the first months of life. Although maternal antibodies acquired transplacentally protect full-term neonates from many systemic pathogens, infections at mucosal surfaces still occur with great frequency, causing significant morbidity and mortality. At least part of this elevated risk is attributable to the neonatal immune system that tends to favor T regulatory and Th2 type responses when microbes are first encountered. Early-life infection with respiratory viruses is of particular interest because such exposures can disrupt normal lung development and increase the risk of chronic respiratory conditions, such as asthma. The immunologic mechanisms that underlie neonatal host–virus interactions that contribute to the subsequent development of asthma have not yet been fully defined. The goals of this review are (1) to outline the differences between the neonatal and adult immune systems and (2) to present murine and human data that support the hypothesis that early-life interactions between the immune system and respiratory viruses can create a lung environment conducive to the development of asthma.

## Introduction

The young of any species face microbial threats similar to those faced by adults. However, the developing mammalian immune system does not mount “adult-type” immune responses to most pathogens. Adaptive in many respects (see below), this immature pattern of response may also contribute to high levels of infection-related mortality and morbidity in young infants. Of the 5 million worldwide deaths in children <5 years of age in 2010, ~64% were due to infectious causes, 40% of which occurred between 1 and 29 days (1). Respiratory infections account for a majority of these neonatal deaths (1). Although bacteria contribute to this disease burden, viral infections are far more common. The annual economic burden of viral respiratory illnesses in the US alone is estimated to be ~$25 billion (2). Many of the respiratory viruses with the greatest impact in young children are well known and include, among others, respiratory syncytial virus (RSV), influenza A/B viruses, rhinoviruses (RVs), human metapneumovirus, parainfluenzaviruses 1–4 (PIV), bocaviruses, coronaviruses, and certain adenovirus strains.

While infant-pattern immune responses may enhance susceptibility to some infections, mounting adult-type responses may not be optimal for overall development either. The neonatal immune system must first and foremost distinguish between self and non-self, and then rapidly proceed to distinguish between benign or even “helpful” non-self (e.g., the commensal microbiome) and potential pathogens. During this period of immune education, the developing immune system has to strike a delicate and pathogen-specific balance between the induction of potentially damaging, pro-inflammatory responses mediated by T helper (Th) cell 1-type (Th1) and some Th17-type cells, less damaging inflammatory responses (e.g., Th2-type) or even suppressive responses mediated by regulatory T cells (Treg). Although the induction of suppressive responses may seem counterintuitive when dealing with potential pathogens, such responses appear to be essential to protect the fetus from reacting too vigorously to maternal antigens (3) or to colonization of the neonatal gut and other body surfaces with the normal microbial flora (4). The “cost” to the infant of mounting an aggressive, pro-inflammatory response can be very high. Early-life inflammation has been associated with a wide range of negative long-term consequences including overall development of the body and the brain, neuro-psychological reactions (e.g., to pain) and patterns of inflammatory response (5, 6) [reviewed in Ref. (7, 8)]. It is, therefore, entirely plausible that early-life experiences with respiratory pathogens can have long-lasting effects on the lung.

A number of epidemiological studies have linked respiratory viral infection during infancy with the later development of asthma [reviewed in Ref. (9–11)]. It is currently unknown whether this association is a direct effect of viral replication in respiratory tissues or results from a virus-induced exacerbation of an underlying predisposition to atopy. However, there is evidence from both human and animal studies to support both hypotheses. The purpose of this review is to examine the immunology of the host–virus interaction during the neonatal period in the context of asthma development. We will first briefly describe how the neonatal immune system differs from that of the adult. Because RSV, influenza, and RVs cause a large proportion of respiratory-tract infections in neonates, we will focus primarily on these three pathogens as models to better understand how early-life infection and antiviral immune responses might contribute to the subsequent development of asthma.

## The Immune System of the Neonate

### Adaptive Immunity in the Neonate

Prior to the 1980s, it was commonly thought that the neonatal immune system was in a state of tolerance (12), as demonstrated by mouse experiments in the 1950s showing the apparent lack of recall responses to antigens injected soon after birth (13). Indeed, neonates were considered by some to be “immunodeficient” (14). Upon discovery of different patterns of T cell response in the mid-1980s [e.g., Th1 vs. Th2 type: reviewed in Ref. (15)], it subsequently became clear that what appeared to be a deficiency in responding to recall antigens was instead a reflection of an intrinsic Th2/Treg bias in neonatal mice (16, 17).

A quarter of a century later, Th1- and Th2-type responses are two of the most well characterized aspects of cellular immunity. The classical Th1 response is characterized by production of the cytokines interferon (IFN)γ and interleukin (IL)-12, whereas Th2 responses are typified by production of IL-4 and IL-13. Th1-type responses tend to be pro-inflammatory in nature, with increases in CD8^+^ T cells, and are most often associated with challenges posed by intracellular organisms, including viruses. Th2 responses typically play a central role in defending against invasive helminths, but are also important in modulating potentially damaging inflammatory responses as well as in driving allergic pathologies (e.g., atopy, asthma). Other more recently recognized patterns of immune response have also been implicated in either promoting or preventing the development or evolution of asthma, including Th17-, Th9-, and Treg. In particular, Th17- and Th9-driven responses may promote exacerbations of severe asthma [reviewed in Ref. (18)] and/or promote allergic airways disease through interactions with Th2 cells (19, 20). Tregs, on the other hand, play a central role in limiting excessive reactivity to self and innocuous allergens (21).

While Th2 skewing may be a natural or default response pattern to microbial insults in the neonate, the neonatal immune system can induce adult-like, Th1/Th2 balanced or even Th1-predominant responses under certain conditions (17, 22, 23). Experimental situations in which such responses can be generated typically require potent Th1 inducers such as CpG motifs found in DNA vaccines (24) and oligonucleotide adjuvants (25, 26), mycobacterial cell wall antigens either in the form of live Bacille Calmette–Guerin (BCG) (27) or complete Freund’s adjuvant (17), or lipid-based adjuvants, such as liposomes (28) and oil-in-water emulsions (29). Several of these powerful stimuli have recently been shown to act *via* ligation of pattern recognition receptors (PRR) [e.g., toll-like receptor (TLR) 9 in the case of CpG motifs] (30). Even when an apparently “balanced” Th1/Th2 response has been generated in neonates, recall responses may still reveal an underlying Th2 bias (31).

The mechanisms by which subsequent antigen recall results in a Th2-biased response are still under active investigation. Neonatal mouse T cells produce significantly higher levels of IL-4 within 48 h of *in vitro* T cell receptor (TCR) stimulation (32) compared to adult T cells, which typically take several days to produce high levels of Th2 cytokines (33). This readiness to produce Th2 cytokines has been attributed to hypomethylation of the Th2 cytokine regulatory region on chromosome 11 in neonatal murine CD4^+^ T cells. A similar state of epigenetic control over Th2 cytokine production has also been observed in human neonatal CD4^+^ T cells (34). Yoshimoto and colleagues have recently shown that epigenetic modifications of the Th2 regulatory regions occur during fetal development in the mouse (35). The Th2 regulatory regions in the 14-day fetal thymus are hypomethylated and adult-like methylation patterns appear only 3–6 days after birth along with a decreased propensity to produce Th2 cytokines. Interestingly, this hypomethylation is restricted to the Th2 regulatory regions, and is not seen in the regulatory regions of the *IFN*γ or *Foxp3* loci. Other factors that could potentially influence Th2-biased immune responses in neonates include relative antigen loads (36), lower total numbers of T cells in neonatal organs (37) and the presence of fetal-origin T cells that are strongly Th2-skewed (38).

Cross-regulation between Th subsets is well known and may be particularly effective in the very young. For example, there is evidence that Th1-type signaling is actively suppressed by Th2 cytokines in the context of both antigen challenge and recall in neonates. Using TCR transgenic mice, Li et al. showed not only that recall responses are primarily Th2-like but that these responses are accompanied by apoptosis of antigen-specific Th1 cells (39). This apoptosis of Th1 cells is driven predominantly by IL-4, is enhanced by IL-10, and occurs only in neonatal T cells (40). Neonatal (but not transplanted adult) Th1 cells co-express IL-13Rα1 and IL-4Rα. Upon exposure to IL-4 after antigen challenge, Th1 cells that carry this heteroreceptor undergo apoptosis (12), resulting in a Th2-biased antigen recall response. IL-13Rα1 upregulation on these Th2 cells is due to relatively low frequencies of IL-12-producing (CD8α^+^ CD4^−^) dendritic cells (DCs) in the neonate- a phenomenon that is reversed by day 6 of life when CD8α^+^ CD4^−^ DCs mature and begin to secrete IL-12 (41). By ~8 days after birth, neonatal murine T cells respond to gradual increases in IL-12 by upregulating IL-12Rβ2 and suppressing IL-13Rα1, contributing to the sustained survival of antigen-specific Th1-type T cells (42).

Although the differences between neonatal and adult T cell responses are particularly striking, the capacity to mount competent humoral responses also varies considerably with age as different “waves” of B cell development occur through early-life (43). For example, antibody responses to T cell independent antigens such as polysaccharide antigens can be very weak during the first years of life. Compared to the adult humoral response to any given antigen, the neonatal response is characterized by generally lower levels of antibodies produced with a delayed onset, less efficient antibody affinity maturation (14) and higher levels of B cell apoptosis (44). Despite these clinical observations, major differences between neonatal and adult B cells have not yet been identified at the molecular level. As a result, the relative “defects” in antibody production for T cell independent antigens are likely attributable to other factors such as delayed appearance of B cells fully competent to handle such antigens or deficiencies in the organization of secondary response sites, such as lymphoid and follicular centers, which develop only a few weeks after birth in mice [reviewed in Ref. (14)].

### Innate Immunity in the Neonate

Striking differences in innate immunity between neonates and adults have also been characterized, particularly with regard to IL-12, a key innate Th1 cytokine. The half-life of IL-12p40 mRNA is decreased in cord blood mononuclear cells and these cells produce far less IL-12 than adult peripheral blood mononuclear cells (PBMC) in response to LPS (45). The addition of IL-12 to cord blood cultures increases Th1-type responses to recall antigens *in vitro* (46), increases the activity of natural killer (NK) cells and enhances IFNγ production (45), and suppresses the induction of IL-13Rα1 (41). However, the administration of supplemental IL-12 to 1-week-old mice *in vivo* is not well tolerated, suppressing weight gain and increasing mortality compared to older mice (47). DCs are the main producers of IL-12 (12) and neonatal mice have fewer splenic DCs than adult animals (48), as well as weaker responses to antigen stimulation *in vitro* and *in vivo* (12). It is interesting that neonatal B cells can suppress both IL-12 production and signaling in neonatal DCs in an IL-10-dependent manner (49, 50). Because DCs are positioned at the nexus of the innate and adaptive immune responses, the characteristics of neonatal DCs are likely central to the relatively weak Th1 responses observed in neonates. Monocyte-derived DCs from human cord blood have decreased markers of activation, as well as lower IL-12p35 mRNA expression upon stimulation with ligands such as LPS, CD40 ligation, or Poly I:C, and are poor inducers of IFNγ from adult T cells (51). Neonatal DCs also produce less IFNα and IFNβ than adult cells (52, 53). Recently, it has been suggested that poor production of IFN by early-life DCs is due, at least in part, to posttranscriptional downregulation of TLR7/9 signaling and increases in the regulatory miRNAs, miR146 and miR155 (54). One important outcome of decreased type 1 IFN signaling would be diminished IFNγ responses in neonates.

Both cell surface and cytoplasmic PRRs, including TLRs, NOD-like receptors, and retinoic acid induced gene I (RIG-I)-like receptors, play important roles in the recognition of common microbial products (e.g., LPS, double stranded RNA) and in triggering immune responses in key innate effector cells such as monocytes and DCs. Responses of cord blood DCs to TLR ligation are both qualitatively and quantitatively different from adult DC responses, despite having similar levels of mRNA (53, 55). Using agonists specific to each TLR, Kollmann et al. (56) studied single cell responses of DCs and monocytes in human cord blood compared to neonatal or adult cells differentiated from PBMC. Overall, the neonatal monocytes and DCs produced more IL-6, IL-23, and IL-1β, which can induce Th17 differentiation/activity, as well as IL-10, a classic immunosuppressive cytokine, but less of the Th1-associated cytokines: IFNα, IFNγ, tumor necrosis factor (TNF)α, and IL-12p70. In another study of early-life responses, the ratio of TLR-induced IL-6/TNFα production by neonatal monocytes was high compared to adult cells. Newborn serum was found to have a similarly skewed ratio of these cytokines (57). Serial measurements in human infants suggest that the ratio of TLR-driven inflammatory (e.g., IFNα, IL-12p70, IFNγ) vs. suppressive/regulatory cytokines (e.g., IL-10) shifts slowly toward “adult” values over the first 2 years of life (58). Several factors likely contribute to the sluggish activity of TLRs in neonatal immune cells, including lower MyD88 levels (59), decreased DNA and co-activator binding of IFN regulatory factor 3 (52), and decreased nuclear translocation of IFN regulatory factor 7 (53). Together, these data suggest that stimulation of innate immune cells in neonates/infants favors Th2/Th17 and possibly Treg differentiation rather than Th1 development. This age-related bias in T cell education could plausibly play a pivotal role in the subsequent development of asthma.

There are still other differences between neonatal and adult innate response capabilities. For example, fetal and the neonatal invariant natural killer T cells (iNKT) cells produce equal amounts of IFNγ and IL-4 upon receptor stimulation in sharp contrast to adult iNKT cells that produce predominantly IFNγ (60). Although NK cells are present in higher numbers in the peripheral blood of neonates compared to adults, they express more inhibitory receptors, have truncated maturation and have lower overall functionality (61). The NK maturation defect in neonates has also recently been linked to transforming growth factor-beta (TGFβ), since murine NK cells engineered to lack TGFβ receptor were capable of fully maturing by 10 days after birth (62). Neonatal macrophages, upon stimulation with LPS and polysaccharide antigens, produce more IL-10 than adult macrophages, and secrete less IL-1β, IL-12, TNFα, probably due to weak TLR signaling (63). Neonatal macrophages are also less responsive to IFNγ due to a defect in STAT1 phosphorylation (64) despite having adult levels of phagocytic function (65). Walk and colleagues studied the expression of a number of inhibitory receptors on neonatal cells, and found increased expression of LAIR-1, CD31, and CD200 on a wide range of innate immune cells including neutrophils, monocytes, and NK cells as well as CD4^+^ and CD8^+^ T cells (66). At least some of the apparent “defects” in neonatal immune cell function can be overcome using specific stimuli either alone or in combination. As mentioned above, TLR8 signaling can drive a strong pro-inflammatory response from neonatal monocytes but can also induce neonatal NK cells to produce adult levels of IFNγ in an IL-12-dependent fashion (67).

### Neonatal Lung Mucosal Immunity

During the postnatal period, the lung continues maturation begun *in utero* and undergoes alveolarization (mice, pnd4-21; humans, 36 weeks preterm to 1–2 years) and microvascular maturation (mice, pnd4-21 mice; humans, 0–3 years) (68, 69). A strong type-2 immune cell bias characterizes the lung mucosa during alveolarization in mice as populations of innate type 2 lymphoid cells (ILC2), mast cells, eosinophils, and basophils increase (69). Furthermore, a large proportion of tissue resident alveolar macrophages, unique populations that are self-maintained throughout life and which develop from fetal liver monocytes beginning at pnd3 in mice (70), express CD206, indicative of a type-2 alternatively activate phenotype (71). Conventional CD11b^+^ neonatal murine lung DCs, though few in number, process antigen more efficiently and more readily express CCR7 (69, 72, 73) than adult DCs and, compared to CD103^+^ DCs, preferentially migrate to the draining (mediastinal) lymph node and promote Th2 responses (69). In mice, lung delivery of house dust mite (HDM) extracts at this early stage of development promotes enhanced allergic airways disease [i.e., increased eosinophil recruitment to the lung, airway hyperresponsiveness (AHR)] (69, 73, 74), in a manner that is dependent upon IL-33 (discussed in detail below) and influenced by preferential differentiation of CD4^+^ T cells to a Th2-type phenotype expressing IL-4, IL-5, and IL-13. In fact, additional studies in mice have investigated canonical T lymphocyte responses in the neonatal vs. adult lung (72). In response to anti-CD3 treatment *in vitro*, CD3^+^, CD4/CD8 double negative T cells, which are present in greater frequency during the neonatal period than in adulthood, more readily express GATA-3 and produce more Th2 cytokines, IL-4 and IL-5 (72). Interestingly, priming of neonatal or adult lung DCs with BCG induced a Th2 response when cocultured with neonatal lymph node T cells, a response that switched to Th1 when cocultured with lymph node T cells from adult mice (72). Altogether, these data highlight the importance the temporal window of the alveolarization stage of lung development to promote rapid, detrimental Th2-type inflammatory responses in the lung.

Compelling new data implicate changes to the lung microbiome in differential regulation of maladaptive type-2 allergic responses between neonates and adults. The predominance of Firmicutes and Gammaproteobacteria in the lung is associated with allergic airways disease in both murine models of asthma (75) and human asthmatics (76) and are the major families that colonize the lung during the alveolar stage of development. These commensals change throughout ontogeny as the lung milieu adapts to harbor Bacteroidetes in adulthood (73). Interestingly, Helios^+^ Treg (CD4^+^Foxp3^+^CD25^+^) cells are abundant in the neonatal lung during alveolarization, but are non-tolerogenic as repeated HDM exposure causes robust eosinophilic airway inflammation, AHR, and mucus production associated with allergic airways disease. Later in life, when the lung microenvironment shifts to support Bacteroidetes, Helios^−^ Tregs are abundant and successfully promote regulatory, antiinflammatory responses thereby providing protection to adult mice from allergic airways disease upon exposure to HDM (73).

The neonatal lung is comprised of type 2 innate immune cells and DCs, which rapidly home to the mediastinal LNs to educate naïve CD4^+^ T cells to develop Th2 responses. Furthermore, the postnatal lung supports a microbiome that promotes allergy and ineffectual Treg responses. Altogether, this type-2 biased neonatal mucosal lung environment has the potential to create a “perfect storm” for asthma development upon exposure to viruses or allergens. How this naturally biased state might synergize with early-life respiratory infections to create a lung microenvironment that favors the development of asthma is discussed in the next section.

## Viral Respiratory Infections and Asthma

Allergic asthma is defined as a chronic inflammatory response to inhaled allergens characterized by intermittent airway obstruction, increased Th2 cytokine production, AHR and mucus production. At least 300 million people worldwide have asthma (77, 78). Despite intensive study, the etiology of asthma is still poorly understood, although a family history of atopy is consistently shown to be an important risk factor [reviewed in Ref. (79, 80)]. Genetics undoubtedly play a pivotal role in the development of asthma but genotype is not fully determinative. Environmental factors influence not only the overall risk of asthma but also the onset and severity of disease. As outlined above, respiratory infections are major causes of short-term morbidity and mortality in the first years of life (81) and viral infections are associated with up to 60% of all asthma exacerbations in the young (82). In addition to this apparent “direct” association with exacerbations (83–85), it is also possible that early-life exposure to specific viruses acts to prime some individuals for development of asthma later in life. Indeed, a growing body of epidemiological evidence suggests that early-life respiratory virus infections predispose infants to the development of asthma (see below). Murine models of both the induction of AHR and the exacerbation of existing airway disease in neonatal and infant animals are beginning to provide some mechanistic understanding of these phenomena. Because RSV, influenza viruses, and RVs are ubiquitous causes of respiratory infection in young infants, these agents will be our primary focus. Table 1 provides an overview of studies linking viral respiratory infections and wheeze or asthma in children.

**Table 1:** Evidence linking respiratory viral infection in infancy and asthma development in childhood.

Causative agent	Conclusion	Reference
Viral infection	In young children (<2 years of age) with a high risk of atopy, both RSV and RV detection in nasal aspirates was associated with asthma development at 5 years of age	(86)
	
	Wheezing in young children (<3 years of age) at a high risk for developing asthma (one parent with asthma or respiratory allergies) that tested positive for RSV and RV, was strongly associated (OR = 10) with asthma at 6 years of age	(87)
	
	A greater number of respiratory infections (viral or bacterial) in young children (< 3 years of age) were associated with asthma development at 7 years of age	(88)

RSV	In a prospective cohort study with matched controls, infants hospitalized with severe bronchiolitis and a family history of atopy/asthma had a greater prevalence of RSV-specific IgG antibodies at the first year follow-up and asthma, atopy at the second year follow-up in comparison to the control group	(89)
	
	In young children (<3 years of age) hospitalized with lower respiratory-tract illness, RSV was an independent risk factor for the development of wheezing at 11, but not at 13 years of age, though no association was found between RSV lower respiratory-tract illness and the development of atopy	(90)
	
	Severe RSV bronchiolitis during infancy was associated with increased prevalence of allergic asthma at 18 years of age (e.g., increased asthma, sensitization to perennial allergens, persistent/relapsing wheeze in association with early allergic sensitization, reduced spirometric function)	(91)
	
	Wheezing in young children (<3 years of age) at a high risk for developing asthma (one parent with asthma or respiratory allergies) that tested positive for RSV was associated (OR = 2.6) with asthma at 6 years of age	(87)
	
	In twins, 3–9 years of age, severe RSV infection does not cause asthma, but rather indicates a genetic predisposition to asthma. Hospital discharge registries and parent-completed questionnaires were fitted to genetic variance components models and direction of causation models	(92)
	
	In a prospective cohort study in twins, 3–9 years of age, hospitalization for RSV infection was associated with asthma shortly after discharge and hospitalization for asthma increased long-term susceptibility to severe RSV infection	(93)
	
	Hospitalization during infancy was associated with the development of childhood asthma: 59% of asthma prevalence in children hospitalized with RSV vs. 6% non-hospitalized (overall comparison estimates—systematic review of 27 articles)	(94)

RV	In young children (<2 years of age) hospitalized for wheezing respiratory illness, RV detection in nasopharyngeal aspirates was associated (OR = 4.14) with asthma development 6 years later in comparison to children that were RV negative	(95)
	
	Wheezing in young children (<3 years of age) at a high risk for developing asthma (one parent has asthma or respiratory allergies) that tested positive for RV was strongly associated (OR = 9.8) with asthma at 6 years of age	(87)
	
	In a prospective population-based surveillance of children <5 years of age, RV group C infected children had a greater prevalence of asthma and a discharge diagnoses of asthma, compared to children testing positive for RV group A (42 vs. 23% and 55 vs. 36%, respectively)	(96)

*OR, odds ratio; RSV, respiratory syncytial virus; RV, rhinovirus*.

### Respiratory Syncytial Virus

#### Background, Prevention, and Treatment

There are two principal antigenic sub-types of RSV (A and B), each with multiple genotypes based on their surface glycoprotein G (97). Although both sub-types can infect humans, most (>85%) symptomatic disease is attributable to type A viruses. These viruses are the leading global cause of serious viral respiratory illness in infants (98, 99). Based on seroepidemiology, nearly 90% of all infants are infected at least once by the age of two. There is no vaccine for RSV and treatment options are limited. Although aerosolized ribavirin may provide marginal benefit in severely ill children, this approach is cumbersome and used infrequently (100). The monthly prophylactic use of a monoclonal antibody that targets the surface fusion (F) glycoprotein (e.g., palivuzimab™ and others) can reduce hospitalization rates by about 50% (101) as well as the total number of wheezing days in the first year of life (102). However, the cost of prophylaxis is so high that this approach is generally restricted to infants at greatest risk of severe disease (preterm infants, infants with immunodeficiency, etc.) [reviewed in Ref. (103)].

#### Epidemiologic Link With Asthma

A broad range of epidemiological data strongly supports the association between early-life RSV-related hospitalization, and the subsequent development of asthma in childhood (90) with effects lasting into early adulthood (91). A recent meta-analysis of infants hospitalized with severe RSV during infancy found a 63% prevalence of asthma in children ≤5 years of age that increased to 92% in children between the ages 5 and 12. After 12 years of age, asthma prevalence in these children falls to 48% but all of these numbers are striking compared to background asthma rates of 1–7% in children with no history of early-life, RSV-associated hospitalization (94). The mechanisms that underlie this strong epidemiologic association are not yet fully understood and it has been argued that hospitalization with RSV is simply a “marker” for children genetically predisposed to asthma as opposed to an environmental risk factor for asthma development (104). In fact, it is likely that both are true to some degree. Given the frequency of RSV infection in the first years of life, hospitalization due to severe infection is relatively rare regardless of genetic background, occurring only when RSV moves into the lower respiratory tract (LRT), causing pneumonia and bronchiolitis (105). In most healthy, full-term babies and infants, RSV infection is limited to the upper respiratory tract and causes only mild-moderate symptoms. In a 10-year study in the US, the rates of RSV-related hospitalization ranged from 48.9 per 1,000 in infants less than 3 months of age to 26 per 1,000 in infants older than 1 year (106). Why some children progress to LRT complications is still unknown, but risk factors include a family history of atopy, preterm birth, congenital heart disease, and low levels of maternal anti-RSV antibodies [reviewed in Ref. (107)].

#### Immunology of RSV-Related Asthma Development

##### RSV Contains Pathogen-Associated Molecular Patterns That Induce Innate Type 2 Cytokines

The tissue tropism of RSV is narrow; restricted to cells of the airway epithelia both *in vitro* and *in vivo*. RSV entry on the apical side of these cells (108) is mediated by the viral attachment (G) and fusion (F) glycoproteins (109) that utilize surface glycosaminoglycans such as heparin and nucleolin (110), respectively, as receptors. As outlined above, a Th2-biased and/or immunosuppressive microenvironment in the lungs is associated with both the development and severity of asthma. There is now considerable evidence that the major RSV surface glycoproteins can directly impact the Th1/Th2 balance in the lung (111). Th2-biased airway inflammatory responses, including Th2 cytokine production and influx of eosinophils into the airways, are induced upon vaccination of BALB/c mice with vaccinia virus expressing the RSV G-protein (112–114). Compelling data implicate IL-9, a cytokine associated with the development of asthma (19) in RSV G-protein-dependent induction of Th2-biased airway inflammation in these mice (115). The RSV F protein may also contribute to the development of a Th2 microenvironment in the lung by signaling through TLR4 (116, 117) and there is strong recent data linking TLR4 genotype to severe RSV disease in humans (118). RSV infection of airway epithelial cells (both immortalized cell lines and primary human cells) *in vitro* increases expression of TLR4 (119) and epithelial cell-specific TLR4 signaling is required for Th2-type responses in the murine lung (120–122). For example, allergic airways disease induced by HDM depends upon airway epithelial cell-specific TLR4 expression and is associated with secretion of several innate cytokines associated with Th2-type responses [e.g., IL-25, IL-33, and thymic stromal lymphopoietin (TSLP)], as well as the classic Th2 cytokines IL-4 and IL-13 (122). RSV infection of bronchial epithelial cells also induces production of TSLP *via* activation of retinoic acid induced gene I (RIG-I) and downstream activation of nuclear factor-κB (123). Importantly, RSV infection of mice sensitized to the model allergen ovalbumin (OVA) leads to an increase in Th2 cytokine production in the lung following OVA challenge suggesting that the RSV effect on Th1/Th2 balance in the respiratory tract is not restricted to viral antigens (124, 125). More recently, prior RSV infection of young mice has been shown to enhance allergic airways disease induced by HDM (126).

##### Innate Type-2 Cytokines Activate Antigen-Presenting Cells

Recognition of the powerful effects of IL-25, IL-33, and TSLP produced by respiratory epithelial cells is an important new element in understanding the potential for respiratory viruses to cause and/or exacerbate asthma. These epithelial-origin cytokines play a pivotal role in both the initiation of Th2 type responses and the progression of allergic diseases (127).

Blockade of IL-25 or the absence of its receptor, IL-17RB, reduces Th2 cytokine production, mucus production, and/or AHR in murine RSV infection and RSV-induced asthma exacerbation models (128). Moreover, in the absence of NK cells, IL-25 secreted from airway epithelial cells upregulates the Notch ligand, Jagged1 on the surface of DCs, which induces the development of an RSV-specific Th2 response (129).

Treatment of murine bone marrow-derived macrophages with exogenous IL-25 or IL-33 can induce production of IL-5 and IL-13 *in vitro* (130). Intraperitoneal treatment of mice with IL-33 results in the differentiation of macrophages from the small intestinal lamina propria into an alternatively activated (M2 or AAM) phenotype in a STAT6-independent fashion. Such peritoneal macrophages are major producers of IL-13 *in vivo*. Furthermore, IL-33 links viral infection, macrophages, and ILC2-dependent production of IL-13 to AHR (131–134) and induces ILC2- and IL-13-dependent DC trafficking to the lymph nodes, promoting Th2 adaptive immunity (135). In the case of RSV infection models, IL-33 gene expression as well as the number of leukocytes expressing the IL-33 receptor (ST2) increase in adult BALB/c mice infected with RSV and interestingly, ST2 blockade decreases lung eosinophil recruitment, IL-13 levels and mucus production, without affecting IFNγ levels (136). ILC2s are an important source of IL-13 secretion post-RSV infection and evidence that they play a role in enhanced disease is provided by data showing that adoptive transfer of lung ILC2s purified from RSV-infected mice 2 h prior to RSV infection dramatically increases production of IL-13 and subsequent eosinophil infiltration (137). Similarly, IL-33 production in the lungs of RSV-infected neonatal mice contributes to ILC2 expansion as well as Th2-biased airway inflammatory responses following RSV re-infection in adults (138). Evidence that IL-33 may contribute to pathogenesis in human disease is provided by data showing that IL-33 levels are increased in nasal aspirates of infants hospitalized with RSV infection (138). Similarly, IL-33 mRNA levels are greater in nasal aspirates of infants hospitalized with RSV bronchiolitis with a family history of atopy compared to those with no such history (139).

Thymic stromal lymphopoietin likely also plays a role in the development of Th2 responses in RSV infection. In rat primary airway epithelial cell cultures, infection with RSV triggers an immediate increase in TSLP mRNA and protein, which induces myeloid DCs to express markers associated with Th2 polarization (140). *Ex vivo* RSV infection of human airway epithelial cells of healthy children and children with asthma results in TSLP induction and has been shown to contribute to Th2 inflammation (123). Infection of human primary bronchial airway epithelial cells also results in upregulation of functional TSLP receptor (TSLPR) on these cells, suggesting a feedback loop in which TSLP binding to its receptor increases production of more TSLP (141). TSLP also acts directly on DCs. Lung DCs, along with alveolar macrophages and ILC2s are among the “first responders” to viral infections and allergen exposure in the lung. The relationship between lung epithelial cells and DCs and the development of asthma has been the subject of several recent reviews (142–144) and provides a framework for understanding how RSV-infected epithelial cells may program lung DCs to promote Th2 immunity. TSLP secreted from epithelial cells infected with RSV (123, 140) induces OX40L expression on the surface of DCs (145) that, in turn, drives T cells toward a Th2 cell phenotype (146). A further link between TSLP and IL-25 in RSV infection is provided by evidence that IL-25 enhances the memory Th2 response induced by TSLP-activated DCs (147). IL-33 also upregulates OX40L surface expression on DCs, effectively promoting Th2 immunity in both allergy and RSV infection models (138, 148). TSLP signaling in ILC2s is also necessary for IL-13 production by these cells as TSLPR KO mice and blockade of TSLP signaling with anti-TSLP neutralizing antibody decrease IL-13 lung protein levels, mucus, AHR and weight loss during RSV infection (149).

In humans, when RSV infects primary myeloid and plasmacytoid-derived DCs (150) from healthy volunteers, the viral G-protein decreases DC activation (151). Suppression of DC maturation has also been observed by the viral non-structural (NS) proteins, NS1 and NS2 (152). Moreover, NS1-dependent activity in RSV-infected DCs suppresses the activation and proliferation of both migratory CD8^+^ T cells (CD103^+^CD8^+^) and Th17 cells while supporting the activation and proliferation of Th2 cytokine-producing CD4^+^ T cells (153). Together, these data suggest that RSV infection of epithelial cells and DCs may act synergistically to elicit a Th2-biased response in both mice and humans. This response pattern is likely accentuated by the already Th2-biased nature of the neonate.

Yet another piece of this puzzle may be the induction by RSV of long-lived, lung-resident macrophage populations. RSV infection in adult mice induces polarization of macrophages toward an AAM (M2) phenotype (126, 154). Similar polarization is observed upon RSV infection of peritoneal macrophages *ex vivo* (154). The classification of macrophages is largely based on cytokine production profiles upon activation and, as is the case with Th1 and Th2 lymphocytes, the cytokines IFNγ and IL-4 appear to play central roles in the development of M1 and M2 macrophages, respectively. Upon activation, M1 macrophages (also called classically activated macrophages or CAM) typically secrete inflammatory cytokines, such as IFNγ, IL-12, and IL-1, whereas M2 macrophages secrete IL-4, IL-13, and IL-10 (155). M2 macrophages play an important role in tissue repair (156, 157) [reviewed in Ref. (158)] mediated, at least in part, by TGFβ and platelet-derived growth factor. Studies in both human infants (159) and neonatal mice (160) have demonstrated that the production of TGFβ is increased upon RSV infection. On the one hand, this increase could reflect an adaptive M2 reparative response to the lung injury caused by RSV infection as shown by Shirey and colleagues (154). On the other hand, over-enthusiastic repair of virally damaged airways could be maladaptive with long-term changes in macrophage phenotype that promote pathologic changes in airway structure and function (131, 134, 161). It is, therefore, interesting that RSV infection of neonatal mice is associated with long-term increases in collagen deposition and airway remodeling (125, 162). Although the precise mechanisms by which RSV infection induces M2 polarization are still unknown, it seems likely that production of TSLP, IL-25, and/or IL-33 by RSV-infected or -exposed respiratory epithelial cells contributes to both M2 differentiation and long-term maintenance of a Th2-biased macrophage population in the lung (123, 130, 163, 164). Furthermore, as noted above, the NS1 and NS2 proteins of RSV inhibit the type-I IFN response in human macrophages (165), which could also contribute to the induction of M2 macrophages.

#### Limitations of These Studies

Much of the evidence outlined above in support of a Th2-biasing effect of RSV in the lung has been obtained using adult mice and human cells/cell lines. Neither of these strategies is ideal. The limitations of cell lines and even primary cells are obvious since these reductionist models cannot reproduce the complexity of *in vivo* host–virus interactions. The adult mouse model of RSV infection also has important drawbacks since adult mice are not susceptible to RSV. This model requires the instillation of high titers of virus (10^6^–10^7^ 50% tissue culture infective dose/mL) directly into the LRT. Furthermore, RSV replication in adult mice is relatively poor and occurs primarily in alveolar pneumocytes rather than the bronchiolar epithelial cells as occurs in humans (166, 167). Perhaps most importantly, RSV infection of adult mice induces a potent Th1 response that rapidly clears the virus. The role of RSV-specific Th2 responses in this model may be restricted to the prevention of exaggerated Th1-mediated lung pathology (154). In the case of human clinical data, it is difficult to resolve the “chicken-and-egg” problem: do children with a genetic propensity to mount Th2 responses and develop asthma suffer more severe early-life RSV or does early and severe RSV infection cause the subsequent development of asthma (83, 168)?

#### Neonatal RSV Infection Model

In an attempt to address these limitations, several groups, including ours, have developed models of RSV infection in neonatal and young mice with re-infection or exposure to allergens later in life (126, 160, 162, 169, 170). These models demonstrate that early-life RSV infection (within 7 days of birth) elicits little IFNγ production in contrast to adult animals (169) and leads to a “pro-asthmatic” phenotype characterized by AHR, mucus production, airway remodeling, and severe disease upon subsequent allergen exposure or live RSV challenge (126, 160, 162, 170). Consistent with these Th2-biased responses, IL-4Rα is increased on pulmonary CD4^+^ T cells following RSV reinfection of adults (171). The exaggerated Th2-inflammatory response upon re-infection is dependent upon the age at initial infection and production of both IL-13 and IL-33 (138, 170) and is enhanced by the presence of anti-RSV IgE antibodies (172). Specifically targeting Th2 responses in the neonate can reduce these exaggerated responses upon adult RSV reinfection. For example, delivery of antisense oligonucleotides targeting the IL-4Rα (173) or a cell penetrating peptide targeting the STAT6 transcription factor (activated by both IL-4 and IL-13) (162) to neonatal mice at the time of RSV infection reduces enhanced disease upon RSV re-infection of adults. Interestingly, human cord blood CD4^+^ T cells also upregulate IL-4Rα upon *ex vivo* RSV stimulation (171). Consistent with these data, development of enhanced disease is T cell dependent in the neonatal challenge- adult rechallenge model. However, reduced inflammatory responses are seen only if CD4^+^ T cells are depleted at the time of adult re-infection but not during the neonatal exposure (174). On the other hand, depletion of CD8^+^ T cells during either the neonatal infection or adult re-infection significantly decreases enhanced disease in mice (174). How CD8^+^ T cells exposed to RSV during neonatal infection promote disease in adult re-infection is unclear, and somewhat paradoxical, given the protective role that CD8^+^ T cells are believed to play in recovery from viral infections. It is also possible that CD8^+^ T cells play slightly different roles in mouse vs. human disease (175). How CD4^+^ and CD8^+^ T cell populations influence the pathogenesis of RSV infection in reinfected adult mice is intriguing and deserves further evaluation. Finally, repeated RSV infection of weanling mice interferes with Treg-mediated tolerance and increases susceptibility to allergic asthma (176).

**Figure 1:**
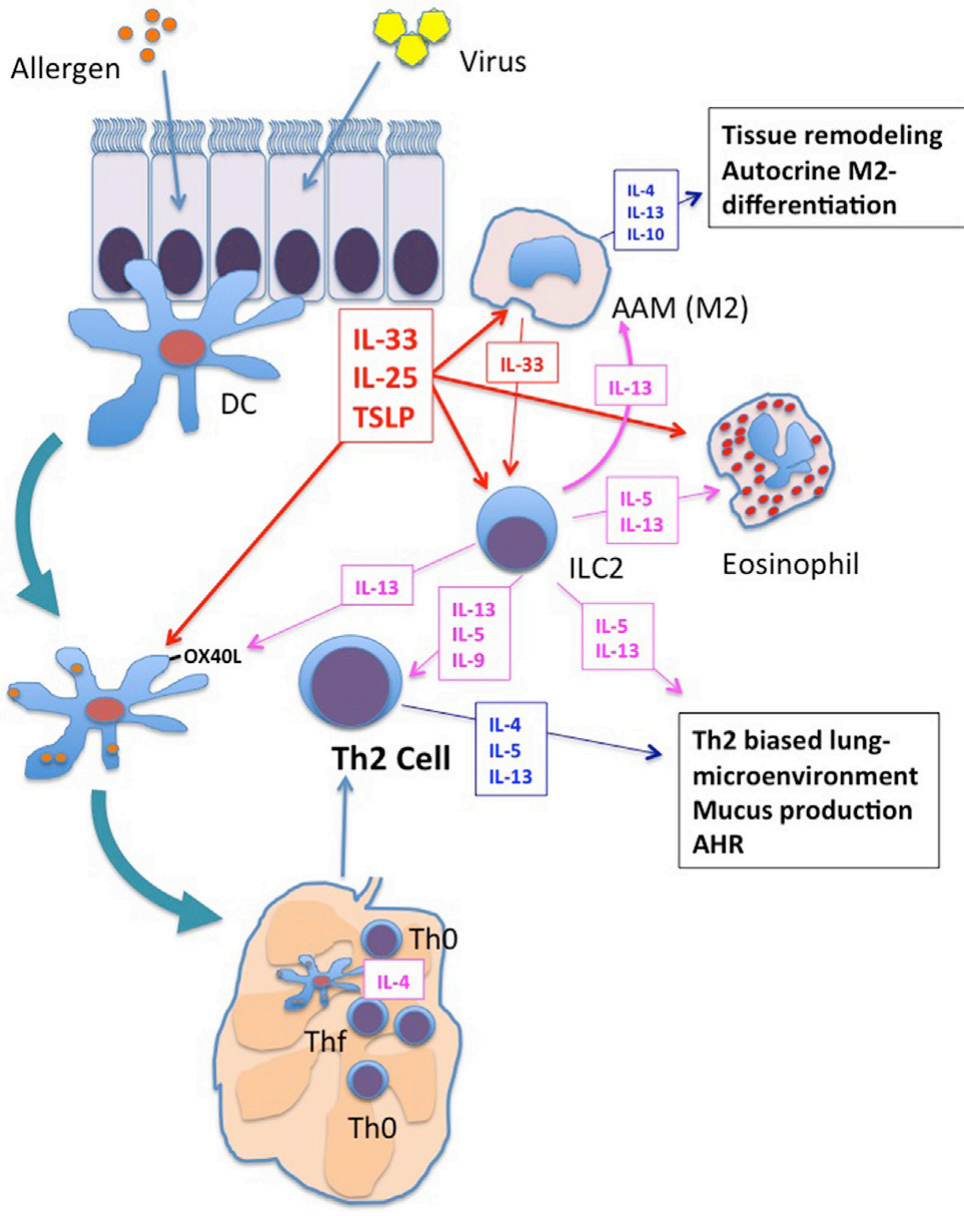
Shared responses in lung innate cells in response to respiratory virus infection and allergen exposure. Viral infection and/or allergen exposure of bronchial epithelial cells results in the secretion of the innate type-2 cytokines: thymic stromal lymphopoietin (TSLP), interleukin (IL)-25, and IL-33. Dendritic cells (DCs), macrophages, and innate type 2 lymphoid cell (ILC2s) are among the first responders in the lung. Each of these cytokines increases activation of DCs, including increased expression of OX40L. OX40L expressing DCs drive Th2 differentiation in the lung mediastinal lymph nodes. These innate type-2 cytokines also promote differentiation of M2 alternatively activated macrophages (AAM), which secrete type-2 cytokines IL-4, IL-13, and IL-10 and orchestrate type-2 inflammation and tissue repair in the lung. M2 macrophage cytokine/chemokine production contributes to enhanced type-2 biased inflammation and airway remodeling. IL-5 and IL-13 production from ILC2s promotes eosinophil influx and responses in DCs and macrophages that further enhance type-2 inflammation, Th2 differentiation, and airway hyperresponsiveness (AHR). Each of these responses is enhanced in neonates upon exposure to respiratory viruses, setting the stage for enhanced type-2 inflammation upon exposure to allergens.

As described in the first section of this review, the neonatal immune system is characterized by a pre-existing Th2 bias. The addition of RSV infection of airway epithelial cells with production of the type-2 innate cytokines, IL-33 (138), TSLP (123), and IL-25 (128), would, therefore, be predicted to create an even more exaggerated type-2-biased microenvironment in the lung with activation of other immune cells (e.g., M2 macrophages, DCs, and/or ILC2 cells) and the development of AHR. When neonatal mice are treated with antibodies to neutralize TSLP, IL-33 or their target expressed on DCs, OX40L, the ability of early RSV infection to prime pathological Th2 responses upon re-infection of adults is reduced (138, 145). As discussed above, TSLP and the other innate type-2 cytokines, IL-25 and IL-33, all promote amplification, differentiation, and maintenance of M2 macrophages (130, 163, 164). Together, these data suggest that many different cells and cytokines, as well as other yet to be determined factors have the potential to contribute to the RSV-induced, Th2 microenvironment and the subsequent development of asthma (Figure 1).

An alternative to the “too much Th2” explanation for Th2-biased neonatal immune system would be “too little Th1”. IFNγ plays a key role in the induction and maintenance of Th1 responses and infection of neonatal mice with recombinant RSV expressing IFNγ prevents enhanced disease upon re-infection (177). A large proportion of the immune cells present in the neonatal mouse lung are macrophages, and these cells are particularly sensitive to stimulation by IFNγ. Indeed, the work of Empey and colleagues shows that IFNγ plays a central role in the balance between the inflammatory M1 macrophage phenotype and the immunosuppressive, M2 phenotype (71). While lung macrophages from adult mice effectively induce M1 macrophage markers in response to RSV, neonatal macrophages require exogenous IFNγ to express M1 markers, produce inflammatory cytokines and drive efficient viral clearance (71). Moreover, administration of intranasal clodronate liposomes that depletes macrophages significantly reduces the ability of IFNγ to promote viral clearance (178). Furthermore, Yamaguchi and colleagues (179) have shown that treatment of neonatal mice with the powerful Th1 adjuvant, CpG, prior to RSV infection prevents enhanced disease upon re-infection, possibly by promoting activation of antigen-presenting cells and production of IFNγ from NK cells. Remarkably, CpG treatment even 4 weeks after early-life infection provides protection (179). These observations raise the possibility that RSV vaccines incorporating CpG or other Th1-biasing adjuvants might be useful not only to prevent infection (180) but also to block the later development of asthma. Whether or not the effects of “more” IFNγ are antigen-specific or simply reflect an adjuvant-modified baseline “potential to respond” in the lung is also an important question. This last possibility is strongly suggested by the work of Remot and colleagues who observed that mucosal vaccination of neonatal mice with nanostructures formed by the RSV nucleoprotein (N) and a combination of Th1-biasing adjuvants (*E. coli* enterotoxin LT and CpG) provides greater protection from immunopathology upon reinfection by RSV later in life compared to nanostructures containing the N protein + LT (181). Together, these data suggest that preventing too much Th2 activity (driven by RSV or other stimuli) or promoting Th1-type activity (or both) may establish a long-term more “balanced” lung microenvironment that resists subsequent development of asthma.

As discussed above, RSV infection inhibits type I IFN production and plasmacytoid dendritic cell (pDC) responses (71, 150, 178).As a result, the maintenance of pDCs during RSV infection may promote beneficial Th1-type responses including the expansion of RSV-specific CD8^+^ T cells. In the challenge–rechallenge model, treatment of neonatal mice during the initial RSV infection with either IFNα or adoptively transferred adult pDCs leads to upregulation of IFNα expression and diminished Th2-biased lung inflammation when these mice are reinfected as adults (182). Further support for the role of pDCs comes from experiments in which adult mice are exposed to FMS-like tyrosine kinase 3 ligand (Flt3-L) prior to RSV infection. In response to Flt3-L, both conventional DC (CD11b^+^CD11c^+^) and pDC (CD11b^−^CD11c^+^B220^+^) populations expand in the lung and draining lymph nodes but only the pDCs protect against airway hyperreactivity, exaggerated Th2 cytokine expression, airway inflammation and mucus production (183). These Flt3-L-induced pDCs have upregulated type I IFN (IFN-α/β) expression and support the expansion of CD8^+^ T cell populations that decrease viral load. Depletion of these pDCs reduces the protective CD8^+^ Th1 response with greater AHR, mucus, viral titers, and Th2 cytokine expression (183, 184). Similarly, Flt3-L administration prior to neonatal RSV infection reduces airway mucus secretion and airway eosinophilia but promotes Th1 RSV-specific CD8^+^ T cells upon adult re-infection (185). Although these observations suggest that Flt3-L protects against RSV infection by inducing pDCs to produce type I IFN, alveolar macrophages (CD11c^+^SiglecF^+^) rather than pDCs (or epithelial cells), are thought to be the main producers of type I IFN in the adult mouse lung (186, 187). Regardless of whether pDCs or alveolar macrophages are the predominant source of type I IFN, these studies strongly suggest that promoting type I IFN production in the lung during early-life RSV infection is likely to lead to adaptive Th1 responses while suppression of type 1 IFN production may favor maladaptive Th2 responses.

Taken together, the data summarized above strongly suggest that RSV infection of the neonatal lung adds a further Th2 influence to an already Th2-biased respiratory microenvironment, with likely contributions from respiratory epithelial cells as well as multiple innate cell populations (e.g., macrophages, DCs, NK cells, ILC2s). The presence of multiple autocrine and paracrine amplification loops between these cells likely leads to the production of excessive IL-4 and IL-13 in the lung that increase both the short- and long-term risk that exposures to otherwise innocuous allergens will lead to immunopathologic responses including asthma (Figure 1).

### Influenza A and B Viruses

#### Background, Prevention, and Treatment

Influenza viruses are far more diverse than RSV due, in large part, to their genetic organization (eight independently segregating genes in the A and B viruses that most commonly infect humans) and the enormous genetic reservoir of influenza viruses in aquatic birds and a number of mammalian species (e.g., most notably pigs but also cats, whales, elephants, skunks, among others) (188). The influenza viruses remain a leading cause of childhood pneumonia globally (189) and early-life infections are common despite the near universal availability of influenza vaccines in resource-rich settings (190). It has been estimated that without vaccination 10–40% of young children are infected by an influenza virus every year (191). Although a number of antivirals are available for influenza infections (e.g., M2 and neuraminidase inhibitors), these drugs have only modest efficacy and their use is further limited by resistance that is either pre-existing or is rapidly induced during treatment (192). Although influenza infections are clearly associated with exacerbations of asthma [reviewed in Ref. (193)], the putative link between early-life influenza and the subsequent development of asthma is much more tenuous. Compared to the large body of work implicating RSV (above), far less work has been done to address this question for influenza viruses.

#### Evidence for a Link Between Influenza Viruses and Asthma

At the current time, there is neither epidemiologic nor experimental evidence in humans suggesting a strong association between early-life influenza infection and asthma. Although work with murine models tends to support an association, these data are not consistent from one model or laboratory to another. Differences in the age of infection, both mouse and virus strain (i.e., human vs. mouse-adapted) and the infective dose may all contribute to these apparently contradictory results. For example, a study by Dahl et al. (194) showed that LRT infection of adult BALB/c mice with influenza A (HKx31 strain) at 6–8 weeks of age could predispose to AHR upon subsequent allergen sensitization (keyhole limpet). Surprisingly, AHR in this model was associated with both Th1-type (IFNγ, IgG2a) and Th2-type (IgG1) antigen-specific responses and the allergic phenotype could be adoptively transferred with pulmonary DCs. Using a similar protocol with OVA as a model allergen in BALB/c mice, Barends et al. found that infection with a mouse-adapted influenza A strain (A/PR/8/34) at the time of OVA challenge decreased Th2 cytokine production in the lungs but increased infiltration by eosinophils (195). Chang et al. have reported that infection of adult BALB/c mice with a reassortant H3N1 influenza A (Mem71) increases alveolar macrophage IL-33 production, driving natural helper cells (i.e., ILC2s) to produce IL-13 that promotes the development of AHR (132). Recently, elevated pleural IL-33 and ILC2s were found to mediate the induction of asthma-like responses (e.g., AHR, production of Th2 cytokines) following pdmH1N1 infection in *Rag1*^−/−^ mice (on a C57Bl/6 background) that lack functional T and B lymphocytes (196).

Like the observations in adult mice, the reported outcomes after early-life influenza A infection are highly variable, sometimes protecting against, but sometimes promoting the development of AHR. When suckling BALB/c mice (2 weeks old) are infected with influenza H3N1 (again Mem71) and challenged with allergens as adults, AHR typically does not occur and the protective effect appears to be mediated by a subset of NKT cells that produce large amounts of IFNγ (197). At high inocula, many influenza viruses are lethal for young mice (198). Using low titer H1N1 virus (PR8), Lines and colleagues found a delayed T cell response with a pronounced influx of eosinophils into the lungs of C57Bl/6J neonates compared to adult mice (198). *Ex vivo* stimulation of lung cells with phorbol 12-myristate 13-acetate/ionomycin in these experiments showed delayed and lower levels of IFNγ in the neonatal immune T cells (198). Others have shown that infection of BALB/c mouse pups with PR8 on day 7 of life can result in long-term pulmonary dysfunction, AHR and an increase in inflammatory cytokines, neutrophils and alveolar macrophages in the lungs (199). In this model, IFNγ was not produced by the neonatal pulmonary CD8^+^ T cells, but adoptive transfer of adult CD8^+^ T cells prevented the long-term AHR (199). Although this body of work is much smaller than that dealing with RSV, the available data raise the possibility that deficient IFNγ production in the neonate during influenza virus infection may play a role in the induction of an asthmatic phenotype upon subsequent exposure to viruses or allergens. Further studies, including the development of a “standard” early-life mouse model of influenza infection are required to begin to understand the potential contribution of these viruses to asthma initiation.

### Rhinoviruses

#### Background, Prevention, and Treatment

Rhinoviruses are the most important etiological agents of the “common cold”. Similar to RSV, RV can cause both upper and lower respiratory-tract illness at all ages. There are 100–150 strains of RV, divided into three groups: A, B, and C. Groups A and B were discovered in the 1990s, while RV-C, a new group with over 50 strains, was identified only in 2006 (200, 201). Most RV infections are thought to be minimally symptomatic or completely asymptomatic. Under 4 years of age, at least one RV can be found by nasal swab in 12–32% of asymptomatic children [reviewed in Ref. (202)]. Although both the scientific literature and the internet offer a wide variety of therapies to prevent or cure the common cold (203, 204), at the current time neither antivirals nor vaccines are available for RV infections.

#### Association of RV With Human Asthma

Similar to RSV, the RVs, and particularly the RV-C group, have been implicated in both the development of asthma and in exacerbation of wheezing illness (87, 96, 205–207). Interestingly, RV-associated wheezing tends to be more common in older infants (>1 year old) in contrast to RSV infection in which the most serious manifestations (i.e., bronchiolitis) occur primarily in those <1 year old (208, 209). However, both RSV and RV are frequently isolated in infants hospitalized with severe respiratory symptoms and wheezing (210). As noted above for RSV, it is very difficult to know if this observation means that children with a genetic propensity to wheeze are more likely to be hospitalized or if RV infections are a causal factor in the onset of wheezing illness (83, 168).

#### Immunology of RV and Asthma

Until recently, animal models suffered from the absence of a receptor for human RV on mouse cells. Most human RV strains bind to intracellular adhesion molecule-1 (ICAM-1) but not mouse ICAM-1 and only a minority (~10%) binds to low-density lipoprotein of both humans and mice (211). Bartlett et al. (212) have described two mouse models of RV infection, one in which BALB/c mice are infected with a low-density lipoprotein-binding isolate and a second based upon transgenic expression of human ICAM-1. Infection of adult mice in the latter model results in a strong Th1 response with abundant IFNγ production as well as exacerbation of allergic responses. To date, the transgenic model has not been used to study RV infection and asthma initiation in neonatal mice. A third model was recently described by Schneider et al. (213) in which 7-day-old BALB/c mice are infected with RV-1B, leading to the development of AHR and mucus production 4 weeks later. Surprisingly, this neonatal infection resulted in the production of IFNγ along with increases in inflammatory cytokines and chemokines, including TNFα, CXCL1, and CXCL2 in the lungs. These observations are very different from the findings in RSV-infected neonates. However, IL-13 was also strongly induced by RV-1B infection in neonatal mice but not in adult mice (214). Analysis of the lungs 35 days after infection showed that a majority of cells producing IL-13 were NKT cells, while CD4 T cells predominantly expressed IFNγ. There was also an increase in M2 macrophages in the lungs, cells that contribute to RV-1B-induced AHR in allergen-sensitized mice infected as adults (215). Neutralizing IL-13 or IL-4R decreased AHR in mice infected as neonates, suggesting that mechanisms similar to those operating in influenza (197) and RSV (138, 170, 173) may also play a role in RV infection. In HDM (*Dermatophagoides farina*)-sensitized BALB/c mice, RV infection decreases IL-10 and increases production of IL-13, RANTES and TNFα as well as eosinophil infiltration into the lungs (216). Th2 cytokines (IL-4, IL-13) increase the expression of the RV receptor (ICAM-1) on human respiratory epithelial (H292) cells (217). Antiviral responses (IFNβ/λ) appear to be decreased in primary bronchial epithelial cells isolated from children with atopy/asthma and are more permissive of RV16 replication (218). Serum IgE levels in these infants are positively correlated with viral RNA levels in *ex vivo*-infected epithelial cells and negatively correlated with RV-induced IFNβ/λ. Other investigators have found a negative correlation between IFN-induction by RV and airway Th2 responses, including eosinophilia and detectable IL-4, in children with a history of asthma both with and without atopy (218). In a human challenge study with RV14, both atopic and non-atopic subjects with low IFNγ/IL-5 ratios had more symptoms and prolonged viral shedding (219).

The dearth of studies on RV infection in neonatal mice makes it hard to draw conclusions about potential mechanisms of RV-mediated asthma initiation. Further work on neonatal models, particularly using the group C strains associated with clinical asthma in infants (87, 96, 205–207), would be useful. Like RSV, RV primarily targets respiratory epithelial cells, although RV infections are typically “patchy” and thus may not cause the same degree of damage as RSV.

Recent investigations of RV infection in both animal models and human studies have focused on the innate type 2 cytokines IL-25, TSLP, and IL-33. TSLP is secreted at a low concentration from primary human bronchial epithelial cells infected *ex vivo* with RV, but rise dramatically with the addition of IL-4 (220). TLSP and IL-33 are also upregulated in bronchial epithelial cells in response to *in vitro* RV infection but even greater increases in IL-25 are seen in cells from asthmatic patients post-RSV infection (221, 222). RV-induced production of IL-33 and/or TSLP blocks OVA-induced inhalational tolerance, with resultant lung eosinophilia and neutrophilia, as well as increased Th2 and decreased Treg cells. Delivery of neutralizing antibodies that target ST2 (the IL-33 receptor) or RV infection of TSLPR knock-out mice both disrupt the ability of RV to block tolerance in this model (222). These data suggest that RV may increase susceptibility to allergic airways disease, by increasing TSLP and/or IL-33, generating a lung environment conducive to immune recognition of normally harmless antigens/allergens. In a murine model of RV-induced asthma exacerbation using OVA, lung levels of IL-25, expressed by epithelial cells and infiltrating immune cells are greatest in mice exposed to both OVA and RV (221). OVA-RV exposed mice have increased levels of IL-4^+^ basophils, IL-4^+^ CD4^+^ T and ICOS^+^ ST2^+^ non-T (ILC2?) cells in the bronchoalveolar lavage (BAL) fluid, as well as enhanced airway eosinophilia and neutrophilia, and exacerbated Th2 cytokine production, all of which are reduced upon delivery of neutralizing IL-25 receptor antibody (α-IL-17RB) prior to RV infection. Interestingly, α-IL-17RB treatment also decreases lung tissue IL-33 and TSLP protein levels. These data suggest that targeting IL-25 receptor may hold therapeutic promise through mitigation of viral- and allergen-induced inflammation promoted by innate type 2 cytokines.

Interleukin-33 is produced by cultured bronchial epithelial and smooth muscle cells when infected with RV (223, 224). In response to *in vivo* infection with RV16, asthmatics have greater viral loads, increased BAL eosinophilia, and greater respiratory symptom scores compared to non-asthmatic controls. These parameters are also associated with reductions in both peak expiratory flow and FEV_1_. Moreover, elevated levels of the Th2 cytokines (IL-4, IL-5, IL-13) as well as IL-33 are present in the BAL fluid of RV16-infected asthmatics. *Ex vivo* incubation of activated (undifferentiated) CD4^+^ T cells (Th0) or ILC2 cells with supernatants from RV-infected bronchial epithelial cells leads to production of IL-5 and IL-13 by both cell types in an IL-33-dependent manner (223). Significantly, production of IL-5 and IL-13 is 100- to 200-fold greater in ILC2 cells compared to CD4^+^ T cells suggesting that IL-33-dependent activation of ILC2 cells during RV infection could play a major role in the development of asthma. Taken together, these data suggest that pronounced IL-33 secretion by smooth muscle cells and/or IL-25, TSLP, and IL-33 secretion by epithelial cells in response to RV infection may skew the lung microenvironment toward Th2-biased allergic airways disease through activation of both innate (e.g., macrophage, DCs, and ILC2 cells) and adaptive immune cells.

## Conclusion

At the current time, a large body of RSV data in both humans and murine models strongly suggest that there is a maladaptive and “asthma-genic” interaction between the Th2-biased nature of the infant immune system and the Th2-promoting effects of the virus itself. It is also very likely that host genetics play an important role in determining both the short-term (e.g., hospitalization) and long-term (e.g., asthma) consequences of this interaction (90, 93). To date, the only factors known to increase susceptibility to RSV-induced asthma are family history of atopy, premature birth, and certain host genetic polymorphisms that are linked with severe disease (111). Whether or not any child, regardless of his/her genetic background can be “made” asthmatic by early-life RSV infection is an important question that cannot be answered at the current time. In favor of the hypothesis that asthmatics are “born” and not “made”, Gern and colleagues have reported bi-directional cytokine responses in cord blood mononuclear cells stimulated with mitogen or viral antigens (e.g., RSV, RV) from children who were later classified as either wheezing vs. non-wheezing at one year of age (225). Whether or not RSV is unique among early childhood respiratory virus exposures with regard to asthma development is also of great interest. The more limited data available suggest that the RV but not influenza virus infection may have effects similar to RSV. Bonnelykke et al. (88) reported that the number of viral or bacterial respiratory infection episodes within the first year of life is a greater predictor of asthma development than the particular viral or bacterial type(s) (88). In contrast to the contribution of respiratory viruses to asthma development, the role of several viruses, including RSV, RV, and influenza viruses, in asthma exacerbations is very clear (9, 84, 206, 226, 227). Of course, neonates and infants are not just exposed to respiratory viruses in the first weeks-months of life. Rather, they experience a wide range of immunologic challenges with both commensal and potentially pathogenic organisms at multiple epithelial sites throughout childhood. For example, Ege and colleagues have shown that children exposed to wide range of microbes early in life, including children that live on farms, have a reduced risk of developing asthma (the hygiene hypothesis) (228) and germ-free mice can more easily be made airway hyperresponsive than their non germ-free littermates (229). A great deal of work remains to be done to fully understand how the ubiquitous respiratory viruses discussed in this review, as well as other early-life microbial exposures, contribute to the development and perpetuation of asthma.

Such an improved understanding is essential to develop strategies to both prevent asthma development and to mitigate the symptoms of asthma once developed. This review has focused on how a small number of viruses may also contribute to the induction of asthma. In particular, we have described the growing evidence that innate immune effector cells may orchestrate early-life events in the lung to “set the stage” for the later development of asthma. Until very recently, the potential asthma-inducing role of respiratory epithelial cells, macrophages, DCs, ILC2s, and NK/NKT cells has been under-appreciated. Although vaccination to protect the very young from early-life respiratory viruses (including maternal immunization) is one possible strategy (230), these new data suggest that modulation of innate responses during early-life viral infections may also be successful. Of course, any strategy that seeks to alter either the antigen-specific or the “overall” immune response pattern of neonates/infants would have to be approached with great caution (e.g., risk of exaggerated Th1 immunopathology, less effective responses to other pathogens or vaccines). As outlined above, the Th2-biased nature of the neonatal immune system is a strategy that has been “proven” by evolution. Nonetheless, given that asthma has now reached essentially “pandemic” proportions, very few questions in clinical medicine are of greater importance. The concepts described in this review raise the possibility of completely novel strategies for dealing with this pandemic.

## Author Contributions

KR, BS, BW, and EF contributed equally to the writing of the manuscript and production of the accompanying figure.

## Conflict of Interest Statement

The authors declare that the research was conducted in the absence of any commercial or financial relationships that could be construed as a potential conflict of interest.

## Abbreviations

AAM, alternatively activated macrophage; AHR, airway hyperresponsiveness; CAM, classically activated macrophage; DC, dendritic cell; HDM, house dust mite; ICAM-1, intracellular adhesion molecule-1; IFN, interferon; IL, interleukin; ILC2, innate type 2 lymphoid cell; KLH, Keyhole limpet (antigen); M1, classically activated macrophage; M2, alternatively activated macrophages; NF-κB, nuclear factor-κB; NLR, NOD-like receptor; NK, natural killer; NS, non-structural (protein); OVA, ovalbumin; pDC, plasmacytoid dendritic cell; PRR, pattern recognition receptor; RLR, RIG-I-like receptor; RSV, respiratory syncytial virus; RV, rhinovirus; TGFβ, transforming growth factor β; Th, T helper; TLR, Toll-like receptor; TNFα, tumor necrosis factor α; Treg, T regulatory; TSLP, thymic stromal lymphopoietin.
